# A review on management of cardiovascular diseases by olive polyphenols

**DOI:** 10.1002/fsn3.1668

**Published:** 2020-08-13

**Authors:** Arshad Mehmood, Muhammad Usman, Prasanna Patil, Lei Zhao, Chengtao Wang

**Affiliations:** ^1^ Beijing Advance Innovation Center for Food Nutrition and Human Health Beijing Technology and Business University Beijing China; ^2^ Beijing Engineering and Technology Research Center of Food Additives School of Food and Chemical Technology Beijing Technology and Business University Beijing China

**Keywords:** atherosclerosis, bioactive compounds, cardiovascular diseases, hypercholesterolemia, hypertension, olive polyphenols, potential mechanism

## Abstract

Noncommunicable diseases have increasingly grown the cause of morbidities and mortalities worldwide. Among them, cardiovascular diseases (CVDs) continue to be the major contributor to deaths. CVDs are common in the urban community population due to the substandard living conditions, which have a significant impact on the healthcare system, and over 23 million human beings are anticipated to suffer from the CVDs before 2030. At the moment, CVD physicians are immediately advancing both primary and secondary prevention modalities in high‐risk populations. The cornerstone of CVD prevention is a healthy lifestyle that is more cost‐effective than the treatments after disease onset. In fact, in the present scenario, comprehensive research conducted on food plant components is potentially efficacious in reducing some highly prevalent CVD risk factors, such as hypercholesterolemia, hypertension, and atherosclerosis. Polyphenols of olive oil (OO), virgin olive oil (VOO), and extra virgin olive oil contribute an essential role for the management of CVDs. Olive oil induces cardioprotective effects due to the presence of a plethora of polyphenolic compounds, for example, oleuropein (OL), tyrosol, and hydroxytyrosol. The present study examines the bioavailability and absorption of major olive bioactive compounds, for instance, oleacein, oleocanthal, OL, and tyrosol. This review also elucidates the snobbish connection of olive polyphenols (OP) and the potential mechanism involved in combating various CVD results taken up from the in vitro and in vivo studies, such as animal and human model studies.

## Graphical abstract

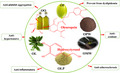

## 1 INTRODUCTION

The noncommunicable diseases account for over 75% of mortalities worldwide particularly in underdeveloped and developing countries and take place almost equally in men and women (Townsend et al., 2016). Cardiovascular diseases (CVDs) are often called “silent killers,” and it is estimated that approximately 17.7 million people died from CVDs representing 31% of all global deaths (Roth, et al., 2017). In addition, CVDs are a group of disorders such as chronic heart disease, stroke, rheumatic heart disease, peripheral arterial disease, congenital heart disease, pulmonary embolism, and deep vein thrombosis and generally proliferate by accumulation of fatty deposits on the inner walls of the blood vessels causing a blockage and stoppage of blood circulation to the arms, legs, brain, and heart (Lanier, Bury, & Richardson, 2016; Roth et al., 2017). Strokes can also be produced by blood loss from a blood vessel. According to the WHO, it is estimated that 7.4 million deaths were due to coronary heart disease and 6.7 million people died due to stroke in 2015. Hypertension is also the most frequent and important intermediate risk factor in addition to diabetes, overweight, obesity, and hyperlipidemia (Roth et al., 2017) Vascular dysfunction has been studied over the past decades, and numerous advances have been made regarding its specific risk factors on the health (Buckland & Gonzalez, 2015). There are two main reasons for vascular diseases, that is, coronary heart diseases and cerebrovascular diseases, such as thromboembolism and atherosclerosis. Atherosclerosis is mainly caused by the accumulation of cholesterol, fat, and other compounds in or on the artery walls, which results in restriction of blood flow to the organs (Mendis et al., 2015). If atherogenesis is not reverted, it will result in smooth muscle cell procreation, production of the extracellular matrix and fibrous tissue, and formation of the necrotic core. Physical disruption of atherosclerotic plaque can cause arterial occlusion, clot formation, and arterial thrombosis (Longo & Mattson, 2014). A healthy lifestyle and diet are fundamental factors for promoting and maintaining good health to decrease the risk of CVDs. The key to good cardiovascular health is to manage the behavioral risk factors such as smoking, less physical activity, harmful use of alcohol, and not as much healthy diet. According to various clinical trials, strategies that are planned to detect and modify the behavioral risk factors can lead to a decrease in hypertension and atherosclerosis development, and also erode the incidence of cardiovascular events. Many medications reduce the risks of CVDs, but most of them exert a wide range of side effects in patients, such as flushing, fatigue, headache, shortness of breath, and dizziness (Blumenthal, Merz, Bittner, & Gluckman, 2008). Among all the behavioral CVD risk factors, a diet is most important and has a huge impact on human health and CVD deaths. The Mediterranean diet (MD) is the heritage of millennia and one of the healthiest diet patterns globally, especially in Mediterranean countries, and eliminates the risk factors for CVDs that represent the principal cause of deaths worldwide. Indeed, MD had been popular in everyday life and declared as an Intangible Cultural Heritage of Humanity by UNESCO in 2010. Traditional MD is considered by the less intake of meat and meat products, more intake of vegetables, fruits, legumes, cereals, and nuts, a low consumption of alcohol, a moderate intake of seafood and fish, and regular use of olive oil (OO), mainly VOO and extra virgin olive oil (EVOO), which intake typically ranges between 25 and 50 ml (approximately two tablespoons) per day. Worldwide cultivation of olive trees (*Olea europaea* L) is more than 8 million ha, and almost 98% of them are cultivated in the Mediterranean basin (Peralbo‐Molina & Luque de Castro, 2013). Spain has the highest number of olive trees and olive orchard (IOC, 2012). In 2014/2015, the total world olive oil (OO) production was 2.39 million tons, and among this, European Union produced 1.53 million tons and it was mainly used for human consumption (IOC, 2014). Besides OO production, olive trees are also used for table olive (TO) production. OO and TO are the two main foods of the MD (Obied et al., 2012). The contemporary world needs natural polyphenols for medications of different types of diseases and increasing interest in the studies of OO and its polyphenols. Therefore, this review discusses the advance research conducted on OO and its polyphenol bioavailability and health benefits with special emphasis regarding the CVDs.

## 2 COMMERCIAL CLASSIFICATION AND CHEMICAL CONTENTS OF OLIVE OIL

Nowadays, OO consumption is abruptly increased on account of the health‐promoting components such as polyphenols, tocopherols and carotenoids (Dias et al., 2018. Further, OO is very important for reducing the growth of foodborne pathogens and stimulation of growth of probiotic microorganisms such as *L. Acidophilus and B. Bifidum*, in addition to antioxidant activity, which make it very popular edible oil in the world (Borges, Alberto, et al., 2017; Borges, Carlos, Alberto, Vique‐Cabrera, & Seiquer, 2017). The classification of OO is dependent on sensory characteristics (aroma, flavor, and off‐flavor) and physicochemical properties (particularly free acidity, peroxide value, and absorption at specific ultraviolet wavelengths). Furthermore, EVOO has good quality due to the maximum presence of physicochemical quality attributes and sensory properties as compared to VOO and LOO. However, VOO has shown off moderate physicochemical quality parameters. The key components of OO are saponifiable lipids (about 98%), which contain mostly triglycerols. The fatty acid present in triglycerols is a monounsaturated fatty acid, particularly oleic acid (55%–83%), which is demonstrated to have numerous health benefits (Ambra, Natella, Lucchetti, Forte, & Pastore, 2017), while saturated fatty acid such as palmitic acid (7.5%–20%) and a polyunsaturated fatty acid such as linoleic acid (2.5%–21%) are also present in the OO (Ramírez‐Tortosa, Granados, & Quiles, 2006). The minor fraction (only 2%) has more than 230 complicated chemical compounds, and it is said to contribute to the organoleptic properties of OO. It also possesses some phenolic compounds (hydrophilic phenols) such as hydroxytyrosol, oleuropein (OL), and tyrosol (Robles‐Almazan et al., 2018). Currently, almost 36 phenolic compounds have been isolated from EVOO and identified, which are present at a wide range of concentrations (0.02–600 mg/kg). In addition, phenolic acids (vanillic acid, syringic acid, gallic acid, etc), flavonoids (eriodictyol, apigenin, luteolin, etc.), secoiridoids (oleacein, oleocanthal, etc.), and lignans ((+) ‐pinoresinol, (+)‐1‐hydroxypinoresinol, (+) 1‐acetoxypinoresinol) are also a part of phenolic compounds (Table 1) (Quirantes‐Pine et al., 2012; Ramírez‐Tortosa et al., 2006). Besides, sterol, 4 methyl sterols, phenolic alcohols, such as triterpenic alcohols, triterpenic dialcohols, fatty alcohols (Srigley, Oles, Reza, Kia, & Mossoba, 2016), hydrocarbons such as carotene, b‐carotene, squalene (Dias et al., 2018; Eggersdorfer & Wyss, 2018), lipophilic phenols, in particular, tocopherols (Borges, Alberto, et al., 2017; Borges, Carlos, et al., 2017), xanthophylls, color pigments (pheophytins, chlorophylls), ketones, waxes, and esters are also a part of the fractions of olive oil (Lombardo, Grasso, Lanciano, Loria, & Monetti, 2018). However, it should be well‐known fact that the composition of more or less 230 complex chemical compounds of OO affected by farming methods, irrigation techniques, climate conditions, geographical regions, variety, and process, that is, extraction, can also affect the chemical contents of OO extract from olive fruit (OF) (Squeo et al., 2016). In case of VOO and EVOO, about 95% of them come from the mesocarp (fleshy mesocarp and epicarp) and only 5% come from the seed of the OF such as embryo and endosperm, after twice pressing, that is, by cold pressing without any chemicals, with small amount of heat applied (Borges, Alberto, et al., 2017; Borges, Carlos, et al., 2017). In another study, the crop year and the density of olive tree affected the chemical composition of the OO obtained from OF (Rodrigues et al., 2018). In this sense, it can be concluded that the strength of the biological effects of OO differs as a result of chemical composition.

**TABLE 1:** Distribution of bioactive compounds present in various parts of olive

Classification	Compounds	OF	VOO	EVOO	OMW	OPW	OL
Phenolic acids	*p*‐coumaric acid	–	0.81 ± 0.40 mg/kg	3.28 ± 0.02 mg/kg	–	0.549 ± 0.038 g of tyrosol	–
	Vanillic acid	0.058 ± 0.01 mg/g	2.00 ± 0.33 mg/kg	–	10.4 ± 0.66 mg/kg	45.0 ± 16.9 lg/g DM	0.002 ± 0.0002 mg/g
	Caffeic acid	2.17 ± 0.02 mg/g	23.8 ± 0.62 mg/kg	0.35 ± 0.00 mg/kg	20.7 ± 0.56 mg/kg	33.3 ± 24.7 lg/g of DM	0.081 ± 0.001 mg/g
	Ferulic acid	–	4.6 ± 0.8 mg/kg	0.01 ± 0.00 mg/kg	7.2 ± 0.08	–	–
	Syringic acid	0.5 6 ± 0.1 mg/kg	15.1 ± 0.74 mg/kg	–	22.4 ± 0.38	0.2 ± 0.1 lg/g of DM	–
Flavonoids	Apigenin	6.1 6 ± 0.6 mg/kg	0.91 ± 0.08 mg/kg	1.72 ± 0.01 mg/kg	–	3.3 ± 1.4 lg/g of DM	–
	Luteolin	0.38 ± 0.06 mg/g	2.68 ± 0.25 mg/kg	15.54 ± 0.13 mg/kg	–	105.1 ± 32.2 lg/g of DM	0.081 ± 0.002 mg/g
	Luteolin−7‐O‐glucoside	0.43 ± 0.03 mg/g	0.43 ± 0.03 mg/g	–	–	32.0 ± 22.1 lg/g of DM	0.008 ± 0.0002 mg/g
	Rutin	4.3 6 ± 0.3 mg/kg	0.002 ± 0.0002 mg/g	–	11.7 ± 0.39%	0.44%TPC	0.13 ± 0.09 mg/g
Secoiridoids	Oleuropein	6.53 ± 0.01 mg/g	140 ± 2.99 mg/kg	–	83.0 ± 3.60 mg/kg	12.4 ± 740.1 lg/g of DM	0.042 ± 0.001 mg/g
	*p*‐HPEA‐EDA	41.1 ± 19.8 mg/kg	59.91 ± 6.95 mg/kg	55.31 ± 0.33 mg/kg	–	3.41 %TPC	–
	3,4‐DHPEA‐EDA	384.0 ± 10.8 mg/kg	75.47 ± 23.91 mg/kg	42.02 ± 0.38 mg/kg	–	–	–
	3,4‐DHPEA‐EA	186 ± 1.2 mg/kg	33.71 ± 1.88 mg/kg	60.22 ± 0.51 mg/kg	–	1.04 %TPC	–
Lignans	(+)‐pinoresinol	–	8.8 ± 0.01 mg/kg	0.42 mg/100 g	–	0.96%TPC	–
	1‐acetoxypinoresinol	–	27.1 ± 1.15 mg/kg 1	–	–	2.3%TPC	–
	Hydroxytyrosol	0.076 ± 0.001 mg/g	41.3 ± 1.04 mg/kg	1.78 ± 0.01 mg/kg	20.7 ± 0.56 mg/kg	1.0 ± 2.5	0.54 ± 0.02 mg/g
	Tyrosol	84.5 ± 6 3.5 mg/kg	23.8 ± 0.62 mg/kg	0.25 ± 0.00 mg/kg	13.5 ± 1.04 mg/kg	282.4 ± 107.5	–
	3,4‐DHPEA‐AC	58.7 ± 0.7 mg/kg	42.29 ± 3.55 mg/kg	0.24 ± 0.00 mg/kg	–	28.4 ± 14.5	–

Note

Adopted from (Ambra et al., 2017; Aludatt et al., 2010; Aggoun et al., 2016; Arslan 2012; Brahim, Kelebek, Ammar, Abichou, & Bouaziz, 2017; Cioffi et al., 2010; El‐Abbassi, Kiai, & Hafidi, 2012; Franco et al., 2014; Leouifoudi et al., 2014; Xie, Huang, Zhang, & Zhang, 2015; Zamora‐Ros, Knaze, & González, 2012).

Abbreviations: DM, Dry matter; EVOO, Extra virgin olive oil; OF, Olive fruit; OL, Olive leaves; OMW, Olive mill water; OPW, Olive pomace waste; TPC, Total phenolic content; VOO, Virgin olive oil.

## 3 OLIVE OIL POLYPHENOL (OOP) BIOAVAILABILITY

At the time of justification of the health benefits of food components, it is essential to consider the difference between bioavailability and absorbed contents of OOP (Difonzo et al., 2017). Altogether during in vitro (cell culture, gastrointestinal digestion) and in vivo (animal and human) investigation, OOP identified was sooner or later got absorbed and was available for storage and physiological events; furthermore, it was also participated in the biological functions quantitatively (Rodriguez‐Concepcion et al., 2018; Mosele et al., 2014). Nowadays, it is considered that in the absorption of lipophilic compounds, in particular, carotenoids (Rodrigues et al., 2018), they are unable to dissolve in the aqueous environment until their release from the matrix, and they need to be merged into colloidal solution. In this sense, the lipids and bile components get developed and possibly picked up by the apical surface of the enterocytes from which lipophilic compounds can be absorbed in a long run, and are further incorporated into circulation (Meléndez‐Martínez et al., 2017). It is noted that the OOPs, for example, hydroxytyrosol and tyrosol, are well reviewed as a bioavailability aspect (Robles‐Almazan et al., 2018; Rosignoli, Fuccelli, Sepporta, & Fabiani, 2016). Moreover, some OOPs such as hydroxytyrosols were properly absorbed in the gastrointestinal tract, but their improper bioavailability resulted in the formation of glucuronide, sulfate, and rapid metabolism (Torre et al., 2008; Mateos et al., 2016). Generally, the low bioavailability of OOP results in an incomplete intestinal absorption and abrupt biotransformation favoring urinary excretion. It is said that the OOP and their secoiridoid derivatives such as oleuropein aglycones, OL, elenolic acid, dialdehydes such as lignans (acetoxypinoresinol), and verbascoside are rapidly hydrolyzed through the gastrointestinal tract. Mode of absorption for hydroxytyrosol and tyrosol is mainly dose‐dependent (Robles‐Almazan et al., 2018). Almost after 1 hr of consumption, their peak plasma level was detected (Miro‐Casas et al., 2003), whereas peak urine concentrations were found after 2 hr of consumption (Miro Casas et al., 2001). The results of in vitro methods (GI and cell culture) also claimed the bioavailability of OOP (Soler et al., 2010; Mosele et al., 2014; Mateos et al., 2016; Pinto et al., 2011; Rosignoli et al., 2016). Provided evidences regarding bioavailability and metabolism of OOP in the human body, animal, GI tract, and cell culture demand further studies that are required to understand the metabolism and bioavailability of OOP.

## 4 OP COMBATING CVDs

**FIGURE 1:**
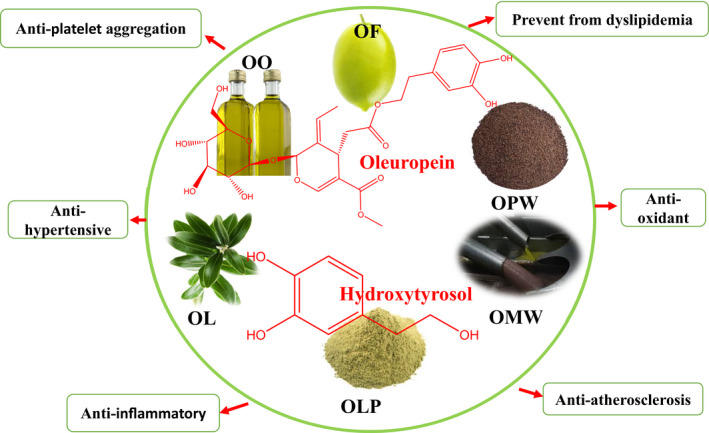
Effects of olive polyphenols on CVDs

This section of the present review describes the studied published olive and its polyphenolic compounds combating CVDs. The summary of key studied results also presented in Table 2 and Figure 1.

**TABLE 2:** Summary of effect of olive polyphenols on CVDs

Disease	Parts used	Study type	Observations	References
Atherosclerosis	OO	In vivo	OO protected from atherosclerosis as compared to saturated fatty acids rich diet in niacin‐treated mice	Montserrat‐de la Paz et al. (2016)
	EVOO	In vivo	EVOO polyphenols improved endothelial function and lowered lipid accumulation within the atherosclerotic lesion of Apo E‐deficient mice.	Claro et al. (2015)
	VOO and thyme	Randomized, double‐blind, crossover, controlled trial	Incorporation of thyme into VOO improved the lipoprotein particle atherogenic ratios in 33 hypercholesterolemic individuals after 3 weeks	Fernández‐Castillejo et al. (2016)
	VOO and thyme	Randomized, double‐blind, crossover, controlled trial	VOO and thyme 25 ml/day for 3 weeks improved endothelial function in 12 healthy subjects	Valls et al. (2017)
	Squalene	In vivo	Administration of squalene to atherosclerotic rabbits reversed endothelial activation and lowered cellularity in gingival mucosa	Bullon et al. (2009)
		In vivo	Administration of hydroxytyrosol at 4 mg/kg bw with presence of saturated fat and cholesterol, reduced the size of atherosclerotic lesions when compared with animals receiving this diet without hydroxytyrosol	Gonzalez‐Santiago et al. (2006)
Platelet aggregation	OO		Hydroxytyrosol and oleuropein inhibited collagen‐induced platelet activation or ADP	Petroni et al. (1995)
			Oleocanthal and oleacein possessed antiplatelet activity by inhibiting COX and 5‐ LOX inhibitor	Beauchamp et al. (2005) and Vougogiannopoulou et al. (2014)
	OOP	In vitro	OLP inhibited platelet function in blood taken from 11 healthy males.	Singh et al. (2008)
	(olive oil polyphenols		OOP inhibited platelet aggregation by cAMP‐PDE inhibition	Dell’Agli et al. (2008)
	EVOO	Randomized crossover	Weekly consumption of EVOO (40 ml) rich in oleocanthal prevented from platelet aggregation.	Agrawal et al. (2017)
Hyperlipidemia	OO		Daily intake of OO (25 ml) didn't promote postprandial lipemia	Weinbrenner, Fitó, et al. (2004)
	(olive polyphenols)	A double‐blind, randomized, placebo‐controlled study	OP (250−1,000 mg for 12 months) to the 64 osteopenic patients women (age: 49 to 68 years) significantly reduced total and LDL cholesterol level	Filip et al. (2015).
	OP	Randomized, crossover, control study	OP significantly increased HDL level in 47 healthy European male volunteers	Hernáez et al. (2014)
	VOO	Double‐blind, randomized, crossover, control trial	VOO (25 ml/day) reduced LDL/HDL particles, HDL cholesterol/HDL‐P ratios, small HDL/large HDL and LP‐IR to the 33 hypercholesterolemic individuals	Ferna´ndez‐Castillejo et al. (2016)
	OLP	human	OLP enrich extract (136 mg oleuropein; 6 mg hydroxytyrosol) for 6 weeks significantly reduced the blood lipid profile in prehypertensive male (60n, aged: 45 years).	Lockyer, Rowland, Spencer, Yaqoob, and Stonehouse (2017)
	OL	A double‐blind, randomized, controlled, longitudinal	OL extract (1.200 mg/day for 28 days) decreased total cholesterol, LDL cholesterol, total cholesterol/HDL cholesterol ratio, oxidized LDL, and GGT in Granada, Spain 39 hypercholesterolemic subjects (aged 45.0 ± 8.8 years)	Fonolla, Diaz‐Ropero, de la Fuente, & Quintela, (2010)
Inflammation	OOP (olive oil polyphenols)		OOP reduced an eicosanoid inflammatory mediators derived from arachidonic acid (thromboxane B2 and 6‐keto‐prostaglandin F1a) and other inflammatory markers, high‐sensibility C‐reactive protein or IL−6	Bogani et al. (2007) and Fito et al. (2008)
	OL	In vitro	Andalusian OL extract inhibited pro‐inflammatory mediator NO in LPS stimulated RAW264.7 cells	Talhaoui et al. (2016)
	EVOO polyphenols		Hydroxytyrosol and tyrosol inhibited MAPK phosphorylation, ROS production and reduced cytokine secretion induced by the oxysterols in PBMCs	Serra et al. (2017)
	OLP (olive leaves polyphenols	double‐blind, randomized, crossover study	OLP (10 mg HT, 51 mg oleuropein) for 4 weeks modulated IL−8 production in 18 healthy volunteers (9 female, 9 male).	Lockyer et al. (2017)
	EVOO		EVOO exhibited inflammatory activity by decreasing NO, ROS production, modulate COX−2, iNOS, and mPGES−1 protein expressions, reduced MAPK phosphorylation and prevented nuclear NFkB translocation in LPS‐stimulated murine macrophages.	C´ardeno et al. (2014)
	OO	Meta‐analysis and systematic review	OO (1–50 mg) decreased C‐reactive protein and interleukin−6	Schwingshackl et al. (2015)
	OLE polyphenols	Double‐blind randomized crossover	OLE polyphenols (6 mg hydroxytyrosol, 136 mg oleuropein) for 6 weeks reduced the interleukin−8 in 60 experimental participants.	Lockyer et al. (2017)
Antihypertensive	OO and VOO		Oleic acid made structure alternations in membrane lipid (H_II_ phase propensity) in that way to handle G protein‐mediated signaling which regulated phospholipase C and adenylyl cyclase, results decreased in BP	Tere´s et al. (2008)
	OO		Oleanolic acid decreased smooth muscle cell Ca by NO release from dependent endothelium and results relaxation.	Rodriguez‐Rodriguez et al. (2008)
	OO	In vivo	OO improved endothelial function in SMRA in SHR rats by modulating an agonist‐mediated EDHF/NO response, which resulted repair dysfunctional endothelium with hypertension	Rodriguez‐Rodriguez et al. (2009)
	OLE polyphenols	Double‐blind randomized crossover	OLE polyphenols (6 mg hydroxytyrosol, 136 mg oleuropein) for 6 weeks reduced BP without the alternation in inflammation, glucose metabolism and vascular function biomarkers in 60 experimental participants.	Lockyer et al. (2017)
	EVOO	Randomized, single‐blind placebo control	Replacing EVOO in American diet reduced BP after 3‐month consumption of EVOO in old overweight/obese (age > 65) participant (41n).	Rozati et al. (2015)
	OO		Polyphenols in OO stimulated NO level which results reduction of BP.	Ferrara et al. (2000)
	OL	In vivo	Oleuropein reduced SBP in male SHR Sprague Dawley rats	Ghibu et al. (2015)
	OL		OL extract EFLA^®^943 (500−1,000 mg) tablets to hypertensive monozygotic twins (40n, age: 16–60), significant reduction in BP was observed after 8 weeks	Perrinjaquet‐Moccetti et al. (2008)
	OL	Human	OL extract EFLA^®^943 (500−1,000 mg twice daily) tablets and Captopril (12.5 mg twice daily) to stage−1 decreased hypertension in patients (aged 25–60 year) after 8 weeks.	Susalit et al. (2011)
	OO	Human	A decrease in BP was observed in stage 1 essential hypertension in young women (24n) when they consumed polyphenol enriched in OO (34 mg/day)	Moreno‐Luna et al. (2012)
	pomace olive oil	In vivo	OPO (10 mg/kg/day bw) to the spontaneously hypertensive SHR rats for 8 weeks modulated endothelial NO	Valero‐Mun˜ oz et al. (2014)
	OO	Meta‐analysis and systematic review	OO consumption (1–50 mg) increased flow‐mediated dilatation and effective for the endothelial function	(Schwingshackl et al., 2015)
	OL	In vivo	OL extract (30 mg/kg/day bw 5 weeks) to SHR rat reduced SBP by modulating the pro‐oxidative and pro‐inflammatory status and improved vascular function.	Romero et al. (2016)
Antioxidant	OO	Human	OO (2.7 to 216 mg/kg) for 14 to 21 days protected from oxidative stress in healthy male participants.	Covas, Nyyssönen, et al. (2006)
	EVOO	Human	EVOO (147 to 592 mg/kg) for 8 weeks prevented from DNA damage in 10 healthy postmenopausal women Florence, Italy	Salvini et al. (2006)
	EVOO	Human	EVOO significantly increased antioxidant enzyme activity CAT, GPX, SOD, decrease in CAT and increase in SOD gene expression was observed	Oliveras‐López et al. (2014)
	EVOO	In vivo	EVOO (330 µl/BW), its lipophilic (3 ml/BW) and hydrophilic (3 ml/BW) fraction for 21 days protected oxidative stress by increasing CAT, GPX, SOD, GSH, NPSH, vitamin C level and decreased plasma LDH, CK, MDA and AOPP level in cardiotoxic rats	Ghorbel et al. (2015)
	Olive leaves (OL).	Cohort, pigs	OL extract 50–100 g/kg bw for 8 weeks to the pigs significantly protected RBCs hemolysis from AAPH or H_2_O_2_ initiators in dose‐dependent manner	Paiva‐Martins et al. (2014)
	olive cake, added with thyme	In vivo	Olive cake, added with thyme extract significantly influenced the plasma and erythrocyte antioxidant status in rat dose‐dependent and time‐dependent manner by inhibiting (DPPH and FRAP), decreased (SOD and GPx) and increased (CAT) level in rats.	Rubió et al. (2014)
	VOO	In vitro	VOO protected from RBCs hemolysis from AAPH or H_2_O_2_ initiators in time (2−4 hr) and dose‐dependent manner (10–80 μM).	Paiva‐Martins et al. (2015)
	EVOO	In vitro	Bioaccessible fractions of EVOO protected from oxidative stress induced by t‐BOOH.	Borges et al. (2015)
	EVOO	In vitro	EVOO increased GSH levels and nonsignificant effects on ROS level.	Kouka et al. (2017)
	VOO	In vitro	VOO showed strong antioxidant activity in ABTS, DPPH, ORAC assays and decreased ROS level in Caco−2 cells.	Quintero‐Florez et al. (2017)

Abbreviations: 5‐ LOX, 5‐lipoxygenase; AAPH, 2,2'‐Azobis (2‐amidinopropane) dihydrochloride, ABTS, 2,2'‐azino‐bis (3‐ethylbenzothiazoline‐6‐sulfonic acid) DPPH, 2,2‐diphenyl‐1‐picrylhydrazyl, ADP, Adenine diphosphate; AOPP Advanced oxidation protein products, BP, Blood pressure; cAMP‐PDE, Cyclic adenosine monophosphate‐phosphodiesterase; CAT, Catalase; CK, Creatine kinase, COX, Cyclooxygenase; EDHF, Endothelium‐derived hyperpolarizing factor; EVOO, Extra virgin olive oil; FRAP, Fluorescence recovery after photobleaching; GGT, Gamma‐glutamyltransferase; GPx, Glutathione peroxidase, GSH, Glutathione, H_2_O_2_, Hydrogen peroxide, HDL, High‐density lipoprotein; IL‐6, Interleukin‐6; iNOS, Inducible nitric oxide synthase; LDH, Lactate dehydrogenase, LDL, Low‐density lipoprotein; LP‐IR, Lipoprotein insulin resistance index; LPS, Lipopolysaccharide; MAPK, Mitogen‐activated protein kinase; MDA, Malondialdehyde, mPGES‐ 1, Microsomal prostaglandin E synthase‐1; NFkB, Nuclear factor kappa‐light‐chain‐enhancer of activated B cells; NO, Nitric oxide; NPSH, Nonprotein thiols, OL, Olive leaves; OLP, Olive leave polyphenols; OO, Olive oil; OOP, Olive oil polyphenols; OP, Olive polyphenols; OPO, Olive pomace oil; ORAC, Oxygen radical absorbance capacity, PBMCs, Peripheral blood mononuclear cells; RBCs, Red blood cells, ROS, Reactive oxygen species; SBP, Systolic blood pressure; SHR, Spontaneously hypertensive; SMRA, Small mesenteric resistance arteries; SOD, Superoxide dismutase, t‐BOOH, t‐butyl hydroperoxide, VOO, Virgin olive oil.

## 5 OP and OXIDATIVE STRESS

The cells in the cardiovascular system contentiously generate reactive oxygen species (ROS) that serve as signaling molecules and also alter or damage the lipids, protein, and DNA, even impair the vascular function and structure (Leopold & Loscalzo, 2009; Lubos, Loscalzo, & Handy, 2011). These ROS are a fundamental part of maintaining cellular homeostatic and work in the Fenton environment, which is balanced by antioxidants (extra‐ or intracellular). It is established that when the ROS level is more than the cellular antioxidant capacity, it produces oxidative stress. At the moment when this ROS level increases, the various kinds of antioxidant system control the ROS level into the cell. These enzymatic and nonenzymatic antioxidants, such as glutathione peroxidases, catalase, superoxide dismutases (reduce the lipid hydroperoxides to lipid hydroxides), ascorbic acid, β‐carotene, α‐tocopherol, and reduced glutathione, play an important role to minimize the oxidative stress. Furthermore, this oxidative stress facilitates atherosclerosis, thrombus formation (oxidizes lipids), and endothelial dysfunction (immune responses and activates inflammation) and eventually results in atherosclerotic plaque formation under several complex mechanisms (Leopold & Loscalzo, 2009; Lubos et al., 2011). The bioactive compounds in virgin olive oil (VOO) alter the postprandial hemostatic profile and protect from oxidative stress (Ruano et al., 2007). A number of studies confirmed that various parts of olive such as OO (Covas, Nyyssönen, et al., 2006), EVOO (Borges, Cabrera‐Viqueb, & Seiquer, 2015; Kouka et al., 2017), VOO (Covas, Nyyssönen, et al., 2006; Quintero‐Florez et al., 2017), leaves (Paiva‐Martins et al., 2014), cake (Rubió et al., 2014), wastewater, and pomace oil have strong antioxidant activity due to the presence of tangible amount of biologically active compounds. Around 50% of biologically active compounds present in olive are hydroxytyrosol and their derivatives as described in Table 2 (Gonzalez‐Santiago, Fonolla, & Lopez‐Huertas, 2010; Raederstorff, 2009). It has been observed that these bioactive compounds are well absorbed even at low concentration (25 ml, 22 g olive oil) in the gut and transferred to bloodstream and protect from ROS formation (Covas, Nyyssönen, et al., 2006; Covas, Ruiz‐Gutiérrez, et al., 2006; Gonzalez‐Santiago et al., 2010; Weinbrenner, Fitó, et al., 2004). However, an earlier study conducted by Vissers, Zock, and Katan (2004) reported that OO did not take a large part in the oxidation process. But, the results of Covas, Nyyssönen, et al. (2006) did not support the previous finding of Vissers et al. (2004). They reported that daily consumption of OO about 2.7–216 mg/kg for 14–21 days prevented healthy male participant from oxidative stress. In another in vivo study, Weinbrenner, Fitó, et al. (2004) documented that OO prevents DNA oxidation. Daily consumption of EVOO (about 147–592 mg/kg) for 8 weeks also prevented DNA damage in 10 healthy postmenopausal women from Florence, Italy (Salvini et al., 2006). One more study by Oliveras‐López, Berná, Jurado‐Ruiz, de la Serrana, and Martín (2014) observed that daily consumption of EVOO polyphenolic‐rich extract significantly improves the plasma antioxidant status in healthy adults. EVOO was daily given (15 ml/day) to healthy adults (45n) for 30 days, and their plasma antioxidant activity (CAT, GPX, SOD) was evaluated. The results revealed that EVOO significantly escalated antioxidant enzyme activity of CAT (42 ± 6–83 ± 13), GPX (90 ± 5–112 ± 6), and SOD (809 ± 191 to 588 ± 146), whereas a decrease in CAT and increase in SOD gene expression were observed without altering other metabolic parameters. Ghorbel et al. (2015) figured out that administration of EVOO (330 µl/BW), and its lipophilic (3 ml/BW) and hydrophilic (3 ml/BW) fractions for 21 days protected oxidative stress by increasing CAT, GPX, SOD, GSH, NPSH, and vitamin C level and decreasing plasma LDH, CK, MDA, and AOPP level in cardiotoxic rats (aluminum trichloride 50 mg//kg bw and acrylamide 20 mg/kg bw). The results of cohort animal studies on pigs also confirmed the strong antioxidant activity of olive leaves (OL). OL extract 50–100 g/kg bw was given to the pigs for 8 weeks. The results demonstrated that OL extracts significantly protected RBCs hemolysis from AAPH or H_2_O_2_ initiators in a dose‐dependent manner (Paiva‐Martins et al., 2014). Additionally, in vivo research conducted by Rubió et al. (2014) reported that olive cake (OC), added with thyme extract, significantly influenced the plasma and erythrocyte antioxidant status in rat. OC, thyme, and their combination (1.5 g/kg bw) were intragastrically gavaged to the rats and elucidated the antioxidant activity of plasma (DPPH and FRAP) and erythrocyte (SOD, CAT, and GPx) assays. The outcomes from that report illustrated that individual and combined extract had a significant effect on plasma and erythrocyte, and antioxidant activity dose depended on time and the manner by inhibiting DPPH and FRAP, decreasing SOD and GPx, and increasing CAT level in rats. Paiva‐Martins et al. (2015) also confirmed that active compounds, that is, 3, 4‐dihydroxyphenylethanolelenolic acid dialdehyde (3, 4‐DHPEA‐EDA) and 3, 4‐dihydroxy phenyl ethanol‐elenolic acid (3, 4‐DHPEA‐EA), in VOO protect from oxidative stress. These compounds also protect from RBC hemolysis and from AAPH or H_2_O_2_ initiators in time‐dependent (2–4 hr) and dose‐dependent (10–80 μM) manner.

The bioaccessible fractions of six Spanish monovarietal EVOO (Arbequina, Cornicabra, Hojiblanca, Manzanilla, Picual, and Picudo) possessed strong antioxidant activity in vitro (ABTS, DPPH, and FRAP) and Caco‐2 cells (Borges et al., 2015). Furthermore, bioaccessible fractions of all varieties except *Picudo* also protect from oxidative stress induced by t‐BOOH. Recently, two studies also supported the previous findings. Kouka et al. (2017) isolated major active compounds in EVOO and investigated antioxidant activity by in vitro assay. The results revealed that EVOO polyphenolic compounds show strong antioxidant activity by increasing GSH levels and have significant effects on the ROS level. However, antioxidant activity was more in hydroxytyrosol as compared to a polyphenolic fraction of EVOO. In another study, Quintero‐Florez et al. (2017) investigated that olive cultivars (Chetoui, Blanqueta, Habichuelero, and Picual) showed strong antioxidant activity in ABTS, DPPH, and ORAC assays. Furthermore, all cultivars of VOO bioaccessible fractions facilitated decreased ROS levels in Caco‐2 cells.

## 6 OP AND HYPERTENSION

Hypertension, which is also known as high blood pressure (HBP), is one of the main risk factors for cardiovascular stroke and myocardial infarction. Hypertension makes structural alternation in the arterial walls of the heart, brain, and kidney. The endothelial dysfunction (decrease in endothelium nitric oxide synthase expression) due to less availability of functional components, oxidative stress (increase in ROS level), inflammation (increase in cytokines production, etc.), and vascular remodeling are the key factors that lead to hypertension. The prevalence of HBP is increasing day by day, and almost one billion people are suffering from this silent killer disease. Therefore, medicines such as angiotensin receptor blockers, angiotensin‐converting enzyme inhibitors, thiazide‐type diuretics, and calcium channel blockers are used to balance the HBP but all caused numerous adverse effects and are out of range due to the expensive cost. Plant‐based medicines are considerable alternative to overcome the cost and less side effect (Armstrong & Joint National Committee, 2014; Turnbull, 2003).

The prophylactic properties of olive (OO, VOO, EVOO, OL, pomace, etc.) are directly associated with the high amount of biologically active compounds such as polyphenols and MUFAs. Recently numerous researchers reported outstrip benefits of consumption of OP toward handling blood pressure (BP), endothelial function in the initial level of hypertension (Ghibu et al., 2015; Lockyer, et al., 2017; Romero et al., 2016; Schwingshackl, Christoph, & Hoffmann, 2015; Valero‐Muñoz et al., 2014). OO and VOO contain around 70%–80% oleic acid, which plays a significant role in the management of BP (Rodriguez‐Rodriguez, Herrera, de Sotomayor, & Ruiz‐Gutierrez, 2007). OO and VOO increase the concentration of oleic acid in the membrane, which makes structure alternations in membrane lipid (H_II_ phase propensity) in a way to handle G protein‐mediated signaling, which regulates phospholipase C and adenylyl cyclase and results in a decrease in BP (Tere´s et al., 2008). A similar mechanism was reported by Rodriguez‐Rodriguez et al. (2008). They reported that OO components (oleanolic acid) decrease smooth muscle cell Ca by NO release from dependent endothelium and result in relaxation. Furthermore, this NO release takes part in PI3K‐dependent phosphorylation of Akt‐Ser^473^ followed by phosphorylation of eNOS at Ser^1177^. In another study, they found that OO improved endothelial function in small mesenteric resistance arteries (SMRA) in spontaneously hypertensive (SHR) rats by modulating an agonist‐mediated EDHF/NO response, which resulted from repair dysfunctional endothelium with hypertension (Rodriguez‐Rodriguez, Herrera, de Sotomayor, & Ruiz‐Gutierrez, 2009). A double‐blind randomized crossover study conducted by Lockyer et al. (2017) reported that consumption of OLE polyphenols (6 mg hydroxytyrosol, 136 mg OL) for 6 weeks reduced BP without the alternation in inflammation, glucose metabolism, and vascular function biomarkers in 60 experimental participants. In another randomized, single‐blinded placebo control study, Rozati et al. (2015) observed that adding EVOO in the American diet (corn, soybean oil, and butter) reduced the BP after 3‐month consumption of EVOO in old overweight/obese (age > 65) contributor (41n). Earlier, Ferrara et al.  (2000) also reported that daily consumption of OO for 6 months reduced the BP significantly in (23n) participants present in the study trial.

Oleuropein is a key biphenol compound present in OL reported to have a strong antihypertensive effect. Oleuropein reduced systolic blood pressure (SBP) in male SHR Sprague Dawley rats (Ghibu et al., 2015). The supplementation of OL extract EFLA^®^943 (500–1,000 mg) tablets to hypertensive monozygotic twins (40n, age: 16–60) showed significant reduction in BP after 8 weeks (Perrinjaquet‐Moccetti et al., 2008). In another comparative study conducted by Susalit et al. (2011), the authors also found similar effects of OL extract EFLA^®^943 (500–1,000 mg twice daily) tablets and captopril (12.5 mg twice daily) to stage 1 hypertension patients (aged 25–60 year) after 8 weeks. A decrease in BP was also observed in stage 1 essential hypertension young women (24n), when they consumed OO (34 mg/day) (Moreno‐Luna et al., 2012). In another study, oral administration of pomace olive oil (POO) (10 mg/kg/day bw) to the SHR for 8 weeks showed antihypertensive effects by modulating endothelial NO (Valero‐Muñoz et al., 2014). Earlier, Rodriguez‐Rodriguez et al. (2007) also figured out that administration (800 ppm, 12 weeks) of POO to SHR rats enhanced eNOS expression and improved endothelial dysfunction. Administration of triterpenic components (oleanolic acid, erythrodiol, maslinic acid, and uvaol) present in POO to SHR rats enhanced eNOS expression and improved the endothelial dysfunction (Rodriguez‐Rodriguez, Perona, Herrera, & Ruiz‐Gutierrez, 2006). A meta‐analysis and systematic review reported that OO consumption (1–50 mg) increased flow‐mediated dilatation and is very effective for the endothelial function (Schwingshackl et al., 2015). Recent study conducted by Romero et al. (2016) also claimed that oral administration of OL extract (30 mg/kg/day bw 5 weeks) to SHR reduces SBP by modulating the pro‐oxidative and pro‐inflammatory status and improves vascular function.

## 7 OP AND INFLAMMATION

Inflammation is responsible for the development of many diseases including CVDs, because release of pro‐inflammatory cytokines such as tumor necrosis factor and interleukin‐1 (IL‐1) in vascular wall directly affects endothelial function by modulating the adhesion molecules and results in endothelial dysfunction, which is a predictor of atherosclerosis and CVDs (Cook‐Mills, Marchese, & Abdala‐Valencia, 2011; Inaba, Chen, & Bergmann, 2010; Roos & Sperber, 1997) . Inflammation and oxidative stress are entangling processes. The bioactive compounds present in OO, EVOO, and by‐products protect from inflammation and endothelial activation (Aparicio‐Soto, Sanchez‐Hidalgo, Rosillo, Castejón, & Alarcon‐de‐la‐Lastra, 2016; Schwingshackl et al., 2015). Regular consumption of OO, butter, or walnut has important effects on the plasma level of pro‐inflammatory cytokines (Jiménez‐Gómez et al., 2009). OOP reduces the eicosanoid inflammatory mediators derived from arachidonic acids, such as thromboxane B2 and 6‐keto‐prostaglandin F1a and other inflammatory markers, such as high‐sensibility C‐reactive protein (hs‐CRP) or IL‐6 (Bogani, Galli, Villa, & Visioli, 2007; Fito et al., 2008). Contradictory results have been obtained concerning the effects of OOP on cell adhesion molecules. A decrease in ICAM‐1 and vascular cell adhesion molecule‐1 serum levels at postprandial state after VOO when compared to refined OO ingestion has been reported (Pacheco et al., 2007). However, no differences in ICAM‐1 levels were reported after sustained virgin or refined OO consumption (Fito et al., 2008). In vitro study conducted by Talhaoui et al. (2016) observed that Andalusian OL extract inhibited pro‐inflammatory mediator NO in LPS‐stimulated RAW264.7 cells and showed anti‐inflammatory properties. EVOO polyphenols (hydroxytyrosol and tyrosol) inhibited mitogen‐activated protein kinase (MAPK) phosphorylation, ROS production, and reduced cytokine secretion induced by the oxysterols in peripheral blood mononuclear cells (Serra, Deiana, Spencer, & Corona, 2017). A double‐blind, randomized, crossover study conducted by Lockyer, Corona, Yaqoob, Spencer, and Rowland (2015) reported that consumption of OLP (olive leaves polyphenols) (10 mg HT, 51 mg OL) for 4 weeks modulated the IL‐8 production in 18 healthy volunteers (nine females and nine males). In another study, Cárdeno, (2014) claimed that EVOO exhibited inflammatory activity by decreasing NO and ROS production. Furthermore, EVOO also modulated COX‐2, iNOS, and mPGES‐1 protein expressions, reduced MAPK phosphorylation, and prevented the nuclear NFkB translocation in LPS‐stimulated murine macrophages. A recent meta‐analysis and systematic review figured out that OO consumption (1–50 mg) decreased in C‐reactive protein and interleukin‐6 (Schwingshackl et al., 2015). A double‐blind randomized crossover study conducted by Lockyer et al. (2017) observed that consumption of olive polyphenols (6 mg hydroxytyrosol, 136 mg OL) for 6 weeks reduced the interleukin‐8 in 60 experimental members.

## 8 OP AND HYPERLIPIDEMIA

Hyperlipidemia is another CVDs referring to an elevated level of fasting and total cholesterol level in the blood. Postprandial lipemia and hyperglycemia are the key risk factors for causing atherosclerosis and other CVDs (Roche & Gibney, 2000). Types and concentration of fat intake also influenced postprandial lipemia. It is reported that the daily intake of OO (25 ml) did not promote postprandial lipemia (Weinbrenner, Fitó, et al., 2004). Because, OO is rich in MUFA, which decreases LDL level and increases HDL. Numerous, in vitro, animal, human, and meta‐analysis reported that OO and its components are helpful in lowering blood lipid profile (Ferna´ndez‐Castillejo et al., 2016; Filip et al., 2015; Fonolla et al., 2010; Hernáez et al., 2014; López, 2008; Lockyer et al. 2017). A double‐blind, randomized, placebo‐controlled study revealed that 12‐month consumption of OP‐enriched extract (250–1,000 mg) by the osteopenic female patients (64n, age: 49–68 years) significantly reduced total and LDL cholesterol level (Filip et al., 2015). Another randomized, crossover, controlled study also elucidated that consumption of OP significantly increased the HDL level in 47 healthy European male volunteers (Hernáez et al., 2014). A recently double‐blind, randomized, crossover, controlled trial also claimed that consumption of VOO (25 ml/day) reduced LDL/HDL particles, HDL cholesterol/HDL‐P ratios, small HDL/large HDL, and lipoprotein insulin resistance index (LP‐IR) in 33 hypercholesterolemic individuals (Fernández‐Castillejo et al., 2016). Lockyer et al. (2017) observed that consumption of OLP‐enriched extract (136 mg OL; 6 mg hydroxytyrosol) for 6 weeks significantly eliminated the blood lipid profile in prehypertensive man (60n, aged: 45 years). Earlier, a double‐blind, randomized, controlled, longitudinal study reported that consumption of OL extract (1,200 mg/day for 28 days) also eroded total cholesterol (−7%), LDL cholesterol (−12%), total cholesterol/HDL cholesterol ratio (8%), oxidized LDL (−13%), and gamma‐ GT (−28%) in Granada, Spain hypercholesterolemic subjects (39n, aged 45.0 ± 8.8 years) (Fonolla et al., 2010).

## 9 OP AND PLATELET AGGREGATION

During the early stage of endothelial disruption, platelets are the first responders of vascular injury and amplify inflammatory process resultant atherosclerotic disease (Nording, Seizer, & Langer, 2015; Rondina, Weyrich, & Zimmerman, 2013). Several facilitator components are responsible for platelet aggregation such as platelet 12‐LOX‐derived 12‐HETE, COX‐derived TXB2, and 15‐LOX‐derived 15‐HETE (Tourdot, Ahmed, & Holinstat, 2013). To prevent platelet aggregation, it is necessary to limit the platelet activation via inhibiting phosphodiesterase, platelet–platelet interactions through glycoprotein IIb/IIIa, cyclooxygenase (COX), and adenosine diphosphate (ADP) receptors (Yousuf & Bhatt, 2011). OO, VOO, EVOO, and their bioactive compounds prevent the platelet aggregation by various pathways. Hydroxytyrosol and OL are potent antiplatelet compounds that are widely distributed in OO inhibit collagen‐induced platelet activation or ADP (Petroni et al., 1995), whereas oleocanthal and oleacein possess antiplatelet activity by inhibiting COX and a 5‐LOX inhibitor (Beauchamp et al., 2005; Vougogiannopoulou et al., 2014). Earlier, an in vitro study conducted by Singh, Mok, Christensen, Turner, and Hawley (2008) observed that OLP inhibited the platelet function in blood taken from 11 healthy males. In another study, OOP inhibited platelet aggregation by cAMP‐PDE inhibition (Dell’Agli et al., 2008). A recently randomized crossover study conducted by Agrawal et al. (2017) reported that weekly consumption of EVOO (40 ml), which is rich in oleocanthal, resulted in the stoppage of the platelet aggregation.

## 10 OP AND ATHEROSCLEROSIS

Atherosclerosis is a systemic lipid‐driven inflammatory condition associated with endothelial dysfunction that results in accumulation and subsequent oxidation of lipids in the vessel wall or plaque development. These abnormalities trigger inflammatory cell infiltration and macrophage foam cell formation, leading to apoptosis and secondary necrosis and plaque advancement (Tabas, 2010). Montserrat‐de la Paz et al. (2016) reported that diets enrich with OO‐protected atherosclerosis as compared to saturated fatty acid‐rich diet in niacin‐treated mice. Another in vivo study conducted by Claro, Ogalla, Rodriguez‐Rodriguez, Herrera, and de Sotomayor (2015) also claimed that EVOO polyphenols lowered the protein expression and macrophage accumulation of iNOS, VCAM‐1, ICAM‐1, NFκB, TNF‐α, and superoxide anion production. Furthermore, EVOO polyphenols improved endothelial function and lowered lipid accumulation within the atherosclerotic lesion of Apo E‐deficient mice. A randomized, double‐blind, crossover, controlled trial, conducted by Fernández‐Castillejo et al. (2016), reported that incorporation of thyme into VOO improved lipoprotein particle atherogenic ratios in 33 hypercholesterolemic individuals after 3 weeks. In the recent past, the research work of Valls et al. (2017) also stated that consumption of thyme and VOO 25 ml/day for three weeks improved endothelial function in 12 healthy subjects. Administration of squalene to atherosclerotic rabbits reversed endothelial activation and lowered cellularity in gingival mucosa (Bullon et al., 2009). In rabbits, administration of hydroxytyrosol at 4 mg/kg body weight in the presence of saturated fat and cholesterol reduced the size of atherosclerotic lesions when compared to animals receiving this diet without hydroxytyrosol (Gonzalez‐Santiago, Martin‐Bautista, Carrero, & Fonolla, 2006). Similarly, many earlier studies reported that consumption of OO, VOO, EVOO, and their bioactive compounds prevented from atherosclerosis (Buus et al., 2011; Graham et al., 2012; Lou‐Bonafonte, Arnal, Navarro, & Osada., 2012; Murie‐Fernandez et al., 2011; Zrelli, Matsuoka, Kitazaki, Zarrouk, & Miyazaki, 2011).

## 11 SAFETY OF OP

Olive and its components do not possess toxicity to humans. An animal model study conducted by Lee‐Huang, Zhang, and Huang (2003) reported that an administration of 1 g/kg bw for 7 days did not cause toxicity to the rats. In another in vitro human cell toxicity study, it was also confirmed that OL extract (1 mg/ml) was not toxic to human cell lines (Petkov & Manolov, 1972). Other studies conducted by various researchers reported that OO and its components were not toxic. OO polyphenols enriched with hydroxytyrosol is safe at the limit of 20 mg/kg daily (Christian et al., 2004; Soni, Burdock, Christian, & Botler, 2006).

## 12 CONCLUSION

Over the past few decades, the use of olive oil and its products in everyday life has a long history of nutrition and therapeutical actions. A large number of study results confirmed that OO and its metabolites are very beneficial for the handling of CVDs through several mechanisms and provide some important nutrimental bioactive compounds besides energy and fat‐soluble vitamins. In the recent past, there was a growing trend to conduct a lot of research work on olive oil polyphenols and results found that olive oil polyphenols increase the HDL level, prevent from oxidative stress, reduce thrombogenic, endothelial dysfunction, BP, and inflammation, and alter gene expression responsible for atherosclerosis process. Furthermore, olive oil still needs clinical efficacy to elucidate the understanding of molecular mechanisms. From this point of view, the ingestion of olive oil and its products needs to be recommended not only due to its beneficial fatty acid profile but also in order to gain advantages from its very important bioactive components that have constructive effects on human health.

## CONFLICT OF INTEREST

The authors declare no conflicts of interest.
